# Metabolic Changes in Synaptosomes in an Animal Model of Schizophrenia Revealed by ^1^H and ^1^H,^13^C NMR Spectroscopy

**DOI:** 10.3390/metabo10020079

**Published:** 2020-02-23

**Authors:** Brian R. Barnett, Fariba Fathi, Paulo Falco Cobra, Sue Y. Yi, Jacqueline M. Anderson, Hamid R. Eghbalnia, John L. Markley, John-Paul J. Yu

**Affiliations:** 1 Neuroscience Training Program, Wisconsin Institutes for Medical Research, University of Wisconsin–Madison, Madison, WI 53705, USA; brianrbarnett@gmail.com (B.R.B.); Syi25@wisc.edu (S.Y.Y.); 2 Biochemistry Department, University of Wisconsin–Madison, Madison, WI 53706, USA; ffathi@wisc.edu (F.F.); paulo@nmrfam.wisc.edu (P.F.C.); heghbalnia@gmail.com (H.R.E.); jmarkley@wisc.edu (J.L.M.); 3 Department of Radiology, University of Wisconsin School of Medicine and Public Health, Madison, WI 53705, USA; anderson.3447@osu.edu; 4 Department of Biomedical Engineering, College of Engineering, University of Wisconsin–Madison, Madison, WI 53706, USA; 5 Department of Psychiatry, University of Wisconsin School of Medicine and Public Health, Madison, WI 53705, USA

**Keywords:** nuclear magnetic resonance spectroscopy, synaptosome, metabolomics, schizophrenia, psychiatric disorder

## Abstract

Synaptosomes are isolated nerve terminals that contain synaptic components, including neurotransmitters, metabolites, adhesion/fusion proteins, and nerve terminal receptors. The essential role of synaptosomes in neurotransmission has stimulated keen interest in understanding both their proteomic and metabolic composition. Mass spectrometric (MS) quantification of synaptosomes has illuminated their proteomic composition, but the determination of the metabolic composition by MS has been met with limited success. In this study, we report a proof-of-concept application of one- and two-dimensional nuclear magnetic resonance (NMR) spectroscopy for analyzing the metabolic composition of synaptosomes. We utilize this approach to compare the metabolic composition synaptosomes from a wild-type rat with that from a newly generated genetic rat model (*Disc1* svΔ2), which qualitatively recapitulates clinically observed early *DISC1* truncations associated with schizophrenia. This study demonstrates the feasibility of using NMR spectroscopy to identify and quantify metabolites within synaptosomal fractions.

## 1. Introduction

Synaptosomes are isolated nerve terminals that contain synaptic components such as neurotransmitters, metabolites, and nerve terminal receptors, and they represent an important component in neurotransmission and synaptic plasticity [1,2,3]. With a growing appreciation of the potential role that synaptic dysfunction plays in neurologic and neuropsychiatric diseases including Alzheimer’s disease, Parkinson’s disease, and schizophrenia, synaptosomes have emerged as an accessible model system for studying synaptic function and synapse biology. A major area of interest has been the metabolic composition of synaptosomes, especially as it relates to compositional differences that may be linked to neuropsychiatric disorders [4,5]. With recent work uncovering evidence of unanticipated genetic [6,7,8], molecular [9], and neurostructural [10,11] similarities of several psychiatric diseases, including autism spectrum disorder (ASD), schizophrenia, bipolar disorder, and major depression, there is renewed interest in understanding the functional biological changes underlying psychiatric illness, especially at the level of the synapse. Reports underscoring this renewed interest are those dissecting the role of serotonin in major depressive disorder and anxiety disorders [12,13,14,15,16,17], along with studies examining perturbations in glutamate homeostasis that may contribute to diverse neurologic and psychiatric illnesses such as major depressive disorder and anxiety disorders, as well as Alzheimer’s disease [18,19].

*DISC1* is a central regulator involved in the network of proteins involved in synapse formation and function. It has attracted research interest as a result of its association with a broad range of neurological and psychiatric disorders. As with other genetic variants that have been shown to confer an increased risk for disease [20], the balanced chromosomal t(1;11)(q42.1;q14.3) translocation of the *DISC1* gene has been implicated in psychiatric illnesses, including schizophrenia. The outsized contribution of *DISC1* in the neuropathogenesis of schizophrenia is largely attributable to its role in early neurodevelopment. Yeast two-hybrid screening has revealed that *DISC1* interacts with a class of proteins that associate with microtubules and their associated complexes at a key developmental time point in neuronal migration and patterning [21,22]. A similar *DISC1* microtubule-associated process has indicated the role of *DISC1* in the radial migration of cortical neurons during cortical development [23]. Several research groups have generated animal models of *Disc1* to study the unique molecular signature of psychiatric disease that arises from this genetic locus [24,25]. To expand the inventory of available translational *Disc1* models, we recently reported a novel rat short genetic variant model of *DISC1* truncation (*Disc1* svΔ2), which lacks exons 2–13 following targeted deletion with CRISPR/Cas9 [26] and thus recapitulates clinically observed early DISC1 truncations associated with schizophrenia [27].

Various biophysical methods have been used to identify and quantify the biochemical composition of synaptosomes to better understand its potential relationship to psychiatric and neurodevelopmental illness. Mass spectrometry (MS) is widely used to analyze the synaptosome and to identify the proteins and neurotransmitters involved in neuropsychiatric disorders [28,29,30,31]. However, MS has several disadvantages that limit its ability to accurately identify and quantify neurotransmitters in synaptosomes, including matrix interference, consistency and reproducibility in sample preparation, matrix inhomogeneity, and low mass resolution and spatial resolution of instruments [32]. High-resolution ^1^H nuclear magnetic resonance (NMR) spectroscopy provides quantitative and reproducible information. Its advantages include low handling and preprocessing time, high reproducibility. The method yields high-throughput metabolic fingerprints and is non-destructive, so that samples can be used for multiple experiments. Although one-dimensional (1D) ^1^H NMR is the most commonly used method in metabolomics studies, it suffers from problems of severe spectral overlap, particularly for signals from compounds present at low abundance. Two-dimensional (2D) NMR methods offer approaches to solving the spectral overlap problems of 1D ^1^H NMR. The presence of proteins in biofluids and organic tissues is one of the main challenges involved in the preparation of samples for sensitive NMR data collection and analysis. The presence of proteins results in broadened NMR signals, thus making it difficult, if not altogether impossible, to identify the signals of small molecules. Protein precipitation by methanol, chloroform, or a mixture of organic solvents is an efficient method to remove the proteins from the system. The use of this method with biological samples as has been reported in the literature [33,34,35,36].

Herein, we describe a proof-of-concept study of the use of nuclear magnetic resonance (NMR) spectroscopy to measure the concentrations of synaptosomal metabolites found in the prefrontal cortex in a *Disc1* svΔ2 rodent model of neuropsychiatric illness. The Syn-PER synaptic protein extraction reagent (Thermo Scientific) was used to minimize the protein content of extracts. This represents, to the best of our knowledge, the first application of NMR toward understanding the biochemical composition of the synaptosome.

## 2. Results

**Figure 1:**
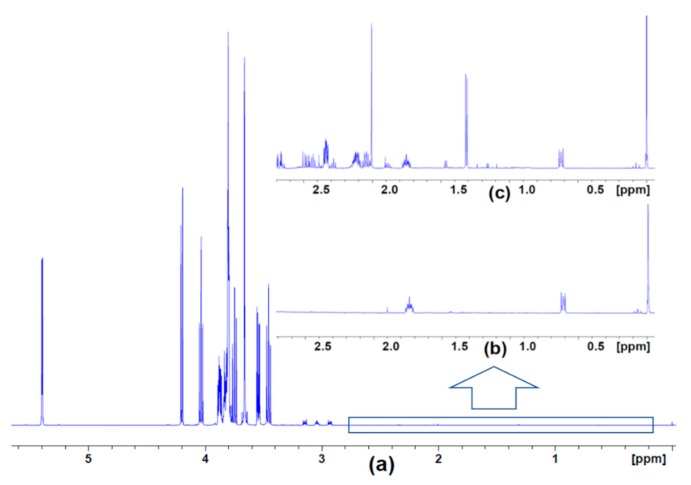
^1^H nuclear magnetic resonance (NMR) spectra of the SYN-Per reagent alone and after its use in extracting metabolites from synaptosomes obtained from control animals. (**a**) Full spectrum of SYN-Per showing the predominant peaks between 3.5 and 4.3 ppm and at 5.4 ppm. (**b**) Expansion of the boxed region of (**a**), which exhibits smaller signals from DSS (sodium trimethylsilylpropanesulfonate) along with the major DSS peak at 0 ppm. (**c**) Same spectral region as in (**b**) following extraction of metabolites from control animals; the additional peaks are ascribed to extracted metabolites.

**Figure 2:**
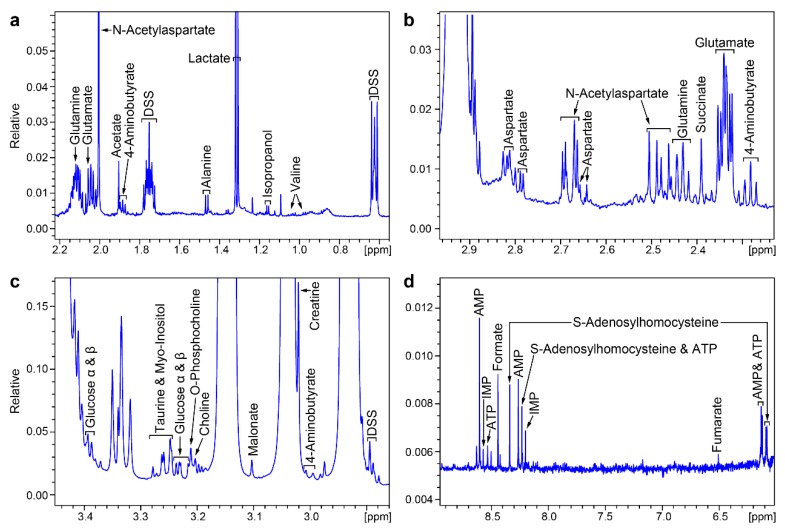
Identification of 20 synaptosomal metabolites in selected regions (**a**–**d**) of the ^1^H NMR spectrum shown in Figure 1.

Although the SYN-Per reagent alone was found to give rise to strong NMR spectral peaks (Figure 1a), when it was used to extract synaptosomes, it was still possible to resolve signals and assign them to extracted metabolites (Figure 1c). We identified signals from a total of 20 metabolites in the ^1^H NMR spectra of extracts from synaptosomes from control rats and Disc1 svΔ2 rats (Figure 2a–d).

**Figure 3:**
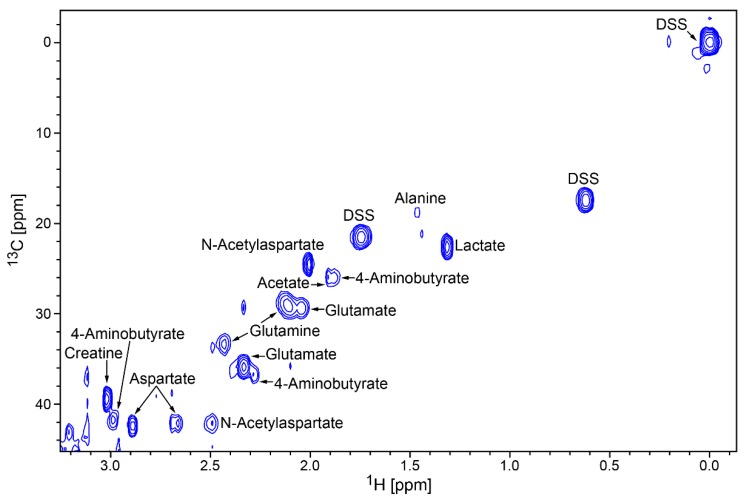
Subset of metabolites identified in the uncrowded region of the 2D ^1^H,^13^C heteronuclear single-quantum correlation (HSQC) spectrum of synaptosomes obtained from control animals. The complete spectrum is provided in the Supplementary Materials (Figure S1).

Noteworthy metabolites and neurotransmitters identified included GABA (4-aminobutyrate), AMP (adenosine monophosphate), ATP (adenosine triphosphate), choline, creatine, glutamate, glutamine, and N-acetyl-aspartate (NAA). The presence of these and other neurotransmitters and metabolites were validated by reference to 2D ^1^H,^13^C heteronuclear single-quantum correlation (HSQC) spectra (Figure 3), which identified several compounds, including AMP, creatine, glutamate, glutamine, and NAA (Table 1).

Among the metabolites in Table 1, nine metabolites were confirmed by 2D ^1^H,^13^C HSQC, as shown in Figure 3. A larger spectral window of the same HSQC spectrum (Figure S1, Supplementary Materials) shows signals assigned to four other metabolites located in a crowded region close to signals from SYN-Per. Because of the low natural abundance of ^13^C (1.1%), peaks from metabolites present in low concentration are often difficult to identify and validate by 2D ^1^H,^13^C NMR. The absence of 2D peaks matching observed 1D ^1^H peaks prevented secondary verification but did not necessarily imply that the metabolites were not present. We next examined metabolite differences between wild-type and Disc1 svΔ2 rats and found trends of higher levels of AMP, aspartate, glutamate, IMP (inosine monophosphate), malonate and NAA in Disc1 svΔ2 rats as compared to wild-type (Table 2; Figure S2, Supplementary Materials).

## 3. Discussion

Recent evidence has pointed to the striking genetic [6,7,8,11], molecular [9] and neurostructural [10] convergence of several psychiatric diseases including ASD, schizophrenia, bipolar disorder, and major depression. While initially arresting, these newly emerging data neatly dovetail into new dimensional frameworks of psychiatric disease on the basis of shared disease comorbidity and neurobiology and bolster the development of the Research Domain Criteria (RDoC) from the National Institute of Mental Health (NIMH). *DISC1* is one such gene that stands at the intersection of numerous psychiatric diseases. As with other genetic variants that have been shown to confer an increased risk for disease [20], the balanced chromosomal t(1;11)(q42.1; q14.3) translocation of the *DISC1* gene has been implicated in several psychiatric illnesses, including schizophrenia and developmental disorders [37,38,39], bipolar disorder [39], autism spectrum disorder (ASD) [40], and major depressive disorder [41]. To further understand the role of *DISC1* in the neuropathogenesis of psychiatric illness, several groups have generated animal models of *Disc1* as an avenue toward understanding its role in the development of the psychiatric disease state. These have included models with dominant-negative *Disc1* expression and models with ENU mutagen-induced point mutations [24,25]. We recently generated a novel CRISPR/Cas9-based *Disc1* svΔ2 biallelic knockout rat model [26] and now utilize this model to explore differences in synaptosome metabolites between wild-type rats and those with this defect.

In the work presented herein, we found evidence of a notable trend toward higher levels of glutamate, aspartate, and NAA in *Disc1* svΔ2 as compared to wild-type, all three of which are critical homeostatic regulators of neurotransmission. Glutamate is the main excitatory neurotransmitter in the central nervous system, and dysfunction of the glutamatergic system has also been implicated in hypotheses of the pathology of several psychiatric disorders such as schizophrenia and ASD [42,43,44]. Aspartate is an N-methyl-D-aspartate receptor (NMDAR) agonist [45], and recent studies have discovered decreases in D-aspartate levels in the prefrontal cortex and striatum of schizophrenic brains and a downregulation of NMDAR subunits [46,47]. Lastly, NAA is a highly concentrated molecule in the brain whose role is still largely unclear. It currently serves as a marker for neurons and oligodendrocytes/myelin, and recent work has suggested that it may also support myelination and play a role in the regulation of neurotransmission [48,49,50].

Interestingly, we also observed a notable increase in concentrations of taurine in *Disc1* animals. Taurine is a naturally occurring sulfur-containing amino acid with broad functional roles in the central nervous system including the modulation of endoplasmic reticulum stress, modulation of apoptosis, and has also been found to prevent the depletion of antioxidant enzymes such as glutathione peroxidase [51]. *Disc1* is known to interact with the mitochondrial proteins mitofilin and CHCDH6 and is also a crucial regulator of mitochondrial trafficking and function [52]. In our *Disc1* svΔ2 animal model, we can infer that the absence of the full length *Disc1* gene product would lead to concomitant deleterious changes in mitochondrial function; that taurine was found to be increased in both male and female animals suggests a compensatory metabolic increase in taurine to potentially counter an increase in reactive oxygen species in the setting of mitochondrial dysfunction.

The application of a multi-dimensional NMR method to analyze the synaptosome represents an important methodological advance. NMR has been a popular method to analyze the metabolic profile of biofluids owing to its reproducibility and its accurate quantitation of low-abundance molecules [53], but has not yet been used for synaptosomes. A multi-dimensional approach utilizing both 1D and 2D NMR can provide a more comprehensive metabolic profile and can also reduce ambiguities in peak identifications [53]. An illustrative example would be for the metabolites creatine and creatinine. Both have peaks at approximately 3 ppm and are difficult to identify, owing to overlapping spectra. Two-dimensional NMR allows us to distinguish between these two metabolites by their ^1^H,^13^C cross-peaks.

A potential limitation of our study was the use of a chemical extraction process. The residual reagent yielded intense broad-shouldered peaks between 3.0 and 4.5 ppm that impeded the analysis of numerous metabolites. Despite this limitation, we were able to definitively identify the 13 salient synaptosomal metabolites enumerated in Table 1 with assignments corroborated by 2D ^1^H,^13^C data. Future work could utilize CsCl ultracentrifugation gradients or further synaptosome purification with HPLC to remove this contaminant. 

Although we found significant differences between the concentrations of certain metabolites from control and *Disc1* svΔ2 synaptosomes, we remain cautious in our interpretation of the quantities and the significance of differences because of possible problems with the extraction protocol and the small sample size: *n* = 6 for each of the four groups.

## 4. Materials and Methods

### 4.1. Animals, Tissue Collection, and Synaptosome Isolation

Twenty-four rats in 4 groups of 6 animals were used for this study; 6 male wild-type animals (control); 6 female wild-type animals (control); 6 male Disc1 svΔ2 knockout animals; 6 female Disc1 svΔ2 knockout animals. The animals were housed and cared for in an AAALAC-accredited facility and the facilities and procedures followed the National Institute of Health’s Guide for the Care and Use of Laboratory Animals. All procedures were approved by the Institutional Animal Care and Use Committee at our institution (M005327, Approved 8 October 2018). Sprague Dawley (SD) rats (300–325 g, Charles River, Worcester, MA, USA) and *Disc1* svΔ2 rats were pair housed in clear cages. *Disc1* svΔ2 animals were generated as previously described [26]. Briefly, utilizing the CRISPR-Cas9 genome-editing technique, the second coding exon of the rat *Disc1* gene encoding amino acids 19–342 (RefSeq transcript ENSRNOT00000057945.4) was targeted for genome editing through the generation of non-synonymous mutations. An in vitro transcription template was generated by overlap-extension PCR with one oligo carrying a 5′ T7 adapter, the target sequence, and a portion of the common gRNA sequence, and the other oligo carrying the antisense common gRNA sequence. The in vitro template was column-purified and in vitro transcribed with the MEGAshortscript kit (ThermoFisher), and the resultant gRNA was cleaned with the MEGAclear kit (ThermoFisher). For injection-grade purification, gRNA was ammonium acetate purified, washed with 70% ethanol, and resuspended in injection buffer. One-cell fertilized Sprague Dawley (SD) embryos were microinjected with a mixture of both gRNAs (25 ng/µL each) and Cas9 protein (PNA Bio, 40 ng/µL), and then implanted into pseudopregnant female Sprague-Dawley (SD) recipients. The resultant pups were genotyped at weaning by PCR, amplifying the targeted region. All animals were maintained under a 12:12 h light:dark cycle in humidity- and temperature-controlled rooms with ad libitum access to water and food.

All animals were acclimated to housing conditions for a minimum of seven days prior to experimental manipulation. At postnatal day 84 (P84), the animals were deeply anesthetized with isoflurane and, following a terminal thoracotomy, the brain was rapidly dissected from the cranial vault and immediately thermally stabilized using a Denator Stabilizor T1 tissue stabilization device (Gothenburg, Sweden), as previously described [54]. All tissues were immediately stored at −80 °C. For synaptosome isolation, the samples were thawed on ice and cerebral tissue from the right neocortex tissue was dissected. For the analysis of the synaptosomal fraction, we used the Syn-PER Reagent (Thermo Fisher Scientific, Waltham, MA, USA) per the manufacturer’s directions. The cerebral tissue was homogenized in Syn-PER reagent, centrifuged at 1500 rpm for 10 min at 4 °C and again at 13,000 rpm for 20 min at 4 °C. The resultant synaptosome pellet was re-suspended in Syn-PER and stored at 4 °C for NMR analysis.

### 4.2. NMR Sample Preparation

To pellet synaptosomal proteins, a 70 µL aliquot of the isolated synaptosome sample was homogenized with 210 µL of ice-cold methanol. The mixture was then vortexed for 30 s and incubated at −20 °C for 20 min and then centrifuged at 13,000 rpm for 30 min at 4 °C. The resultant supernatant was then decanted into fresh vials and dried using a speed vacuum concentrator. The dried samples were solubilized in 70 µL of D_2_O containing 100 mM sodium phosphate buffer (pH 7.4), 0.5 mM DSS and 0.4 % (m/m) NaN_3_. All samples were then transferred to 1.7 mm NMR tubes and stored at −20 °C prior to NMR data acquisition. To increase the signal to noise ratio, 6 individual synaptosome samples were combined. After drying, the combined sample was solubilized D_2_O for HSQC experiment.

### 4.3. NMR Data Collection and Analysis

All spectra were recorded at 298 K using a Bruker Avance III 600 spectrometer (Operating at 600.08 MHz for ^1^H) equipped with a 1.7 mm cryogenic probe. A standard 1D *CPMG* (Carr–Purcell–Meiboom–Gill) pulse program was selected for ^1^H NMR. Spectra were acquired averaging 1024 transients with 24,574 points, acquisition time (AQ) of 1.70 s, and repetition delay of 2 s between transients. The chemical shift in the DSS signal was used as the internal chemical shift standard, and the peak width of this signal in all samples was less than 1 Hz. The relative concentrations of metabolites were obtained by the target profile method, using Chenomx software (NMR suite version 8.3, Edmonton, Alberta, Canada). Chenomx delivers tools for manual determination of metabolite concentration by expert users (including metabolites with overlapped peaks). First, Chenomx fits the experimental NMR chemical shifts and peak shapes (singlets, doublets, triplets etc) to spectra of standard metabolite compounds that are stored in the Chenomx database. Then, Chenomx uses the standard spectral templates to measure the relative concentration of metabolites with respect to DSS (a standard with known concentration). Two-dimensional ^1^H,^13^C heteronuclear single-quantum correlation (HSQC) spectra were also acquired by using a gradient-selected, sensitivity-enhanced pulse program. Each time-domain spectrum of the HSQC experiment was the average of 352 transients consisting of 4096 points with a 1.5 s repetition delay; the second dimension was derived from 256 increments. The spectral widths were 16 ppm and 200 ppm for the ^1^H and ^13^C dimensions, respectively.

## 5. Conclusions

In this study, we have demonstrated the potential utility of multi-dimensional 1D and 2D NMR spectroscopy for the characterization of the biological composition of synaptosomes. In this pilot and proof-of-concept study, we found differences in the metabolic compositions of synaptosomes from wild-type rats and a novel *Disc1* svΔ2 rat animal model of psychiatric illness. We identified 20 metabolites in the ^1^H NMR spectra and confirmed 13 of these by 2D ^1^H,^13^C HSQC spectra. This study demonstrates the potential utility of multi-dimensional NMR to identify and quantify metabolites within synaptosomal fractions. Lastly, this work introduces new experimental avenues to examine the biochemical, biomolecular, and metabolic composition of synaptosomes associated with neurologic and neuropsychiatric disorders of the brain.

## Figures and Tables

**Table 1:** Chemical shifts and multiplicities of ^1^H NMR signals assigned to compounds in extracts of synaptosomes from control animals.

Compound Name	Chemical Shifts and Multiplicities	Notes
4-Aminobutyrate (GABA)	1.9 m, 2.3 t, 3 t	Confirmed by HSQC
AMP	4.01 m, 4.36 m, 4.50 q, 4.79 t, 6.12 d, 8.25 s, 8.58 s	Concentration too low to detect by HSQC
ATP	4.23 m, 4.27 m, 4.56 t, 4.73 t, 6.12 d, 8.24 s, 8.49 s	Concentration too low to detect by HSQC
Acetate	1.9 s	Confirmed by HSQC
Alanine	1.47 d,3.78 q	Confirmed by HSQC
Aspartate	2.66 dd, 2.80 dd, 3.91 dd	Confirmed by HSQC
Choline	3.19 s, 3.50 dd, 4.05 t	Overlapped by SYN-PER peaks
Creatine	3.02 s, 3.91 s	Confirmed by HSQC
Formate	8.44 s	Concentration too low to detect by HSQC
Glucose	3.25 m, 3.42 m, 3.49 m, 3.50 m, 3.54 m, 3.72 m, 3.73 m, 3.77 m, 3.87 m, 3.88 m, 4.66 d, 5.23 d	Confirmed by HSQC; close to SYN-PER area
Glutamate	2.04 m, 2.12 m, 2.32 m, 2.32 m, 3.76 dd	Confirmed by HSQC
Glutamine	2.15 m, 2.18 m, 2.42 m, 2.46 m, 3.76 t	Confirmed by HSQC
IMP	8.53 s, 8.21 s, 6.13 d, 4.49 t, 4.36 m, 4.03 m	Concentration too low to detect by HSQC
Lactate	1.31 d, 4.10 q	Confirmed by HSQC
N-Acetylaspartate	2.1 s, 2.5 dd, 2.7 dd, 4.4 m	Confirmed by HSQC
O-Phosphocholine	3.21 s, 3.58 m, 4.16 m	Confirmed by HSQC; close to SYN-PER area
S-Adenosyl-homocysteine	2.1 m, 2.7 t, 3.0 q, 3.1 q, 3.8 q, 4.3 m, 4.4 t, 4.9 t, 8.10 d, 8.26 s, 8.33 s	Concentration too low to detect by HSQC
Succinate	2.39 s	Concentration too low to detect by HSQC
Taurine	3.26 t, 3.43 t	Confirmed by HSQC; close to SYN-PER area
Myo-Inositol	3.3 t, 3.5 dd, 3.6 t, 4.1 t	Confirmed by HSQC; close to SYN-PER area

**Table 2:** *Disc1 svΔ2* contributes to significant changes in metabolite concentrations in both male and female subjects.

Metabolite ^1^	Mean (*mM*)		*p*-Value	Mean (*mM*)		*p*-Value
	Control Male	*Disc1* svΔ2 Male	Male	Control Female	*Disc1* svΔ2 Female	Female
GABA	0.08980	0.07940	0.66	0.06698	0.07175	0.79
AMP	0.01140	0.03762	0.01	0.01423	0.03068	0.02
ATP	0.00613	0.00757	0.46	0.00545	0.00638	0.38
Acetate	0.03620	0.03483	0.78	0.03195	0.03393	0.10
Alanine	0.02308	0.03030	0.38	0.01948	0.02625	0.23
Aspartate	0.09735	0.13747	0.19	0.07712	0.12468	0.03
Choline	0.01995	0.01900	0.87	0.01525	0.01517	0.98
Creatine	0.33330	0.49193	0.13	0.28313	0.41097	0.07
Formate	0.02252	0.02087	0.31	0.02390	0.02177	0.57
Glucose	0.40657	0.42493	0.30	0.42541	0.42785	0.94
Glutamate	0.37868	0.65835	0.07	0.34222	0.58792	0.04
Glutamine	0.18277	0.24677	0.24	0.14740	0.21837	0.11
IMP	0.00325	0.00815	0.04	0.00445	0.00673	0.10
Lactate	0.24647	0.34892	0.16	0.20898	0.29948	0.09
N-Acetyl-aspartate	0.21753	0.36463	0.08	0.19398	0.32667	0.04
O-Phospho-choline	0.01983	0.02862	0.14	0.01772	0.02702	0.14
S-Adenosyl-homo-cysteine	0.01528	0.01585	0.91	0.01328	0.01383	0.87
Succinate	0.01125	0.01980	0.06	0.01247	0.01703	0.14
Taurine	0.18560	0.29490	0.11	0.15967	0.25010	0.06
myo-Inositol	0.22202	0.33798	0.09	0.21327	0.27798	0.18

^1^ Metabolite concentrations from synaptosomes obtained from Disc1 svΔ2 and wild-type male and female subjects. Calculated *p*-values are presented for illustration purposes (*n* = 6 for all groups; 6 male control; 6 female control; 6 male wild-type and Disc1 svΔ2 knockout rats; 6 male wild-type and Disc1 svΔ2 knockout rats).

## Supplementary Materials

The following are available online at https://www.mdpi.com/2218-1989/10/2/79/s1, Figure S1: Extended view of Figure 3 showing metabolites identified by the 2D ^1^H,^13^C HSQC spectrum of synaptosomes obtained from control animals, Figure S2: Peaks assigned to IMP and ATP in the 1D spectrum obtained for an animal in the control group can be clearly identified. The peaks for IMP and ATP are the weakest peaks in the spectrum with areas approximately one-half those of the next weakest peaks (succinate and homo-cysteine). The concentrations of ATP and IMP are estimated to be about 5 μM.
