# Supplementary material for: Metabolic Changes in Synaptosomes in an Animal Model of Schizophrenia Revealed by 1H and 1H,13C NMR Spectroscopy
Source: Metabolites. 2020 Feb 23;10(2):79. doi: 10.3390/metabo10020079 (PMC7074231; doi:10.3390/metabo10020079)
Supplement: Supplementary file 1 [file metabolites-10-00079-s001.pdf]

Supplementary Materials:

The two figures contained in this supplementary file provide additional information in support of the results in the manuscript. Figure S1 is an expanded view of manuscript Figure 3. In addition to metabolites identified in Figure 3, Figure S1 identifies additional peaks in the more crowded and noisier region of the spectrum that we have assigned to metabolites. Figure S2 exhibits the spectral region of the 1D ^1H spectrum of the synaptosome from a control animal that contains peaks from IMP and ATP. Although these peaks correspond to metabolites with the lowest measurable concentration, their peak shapes are clearly distinguishable and enable measurement of the areas under the peaks.

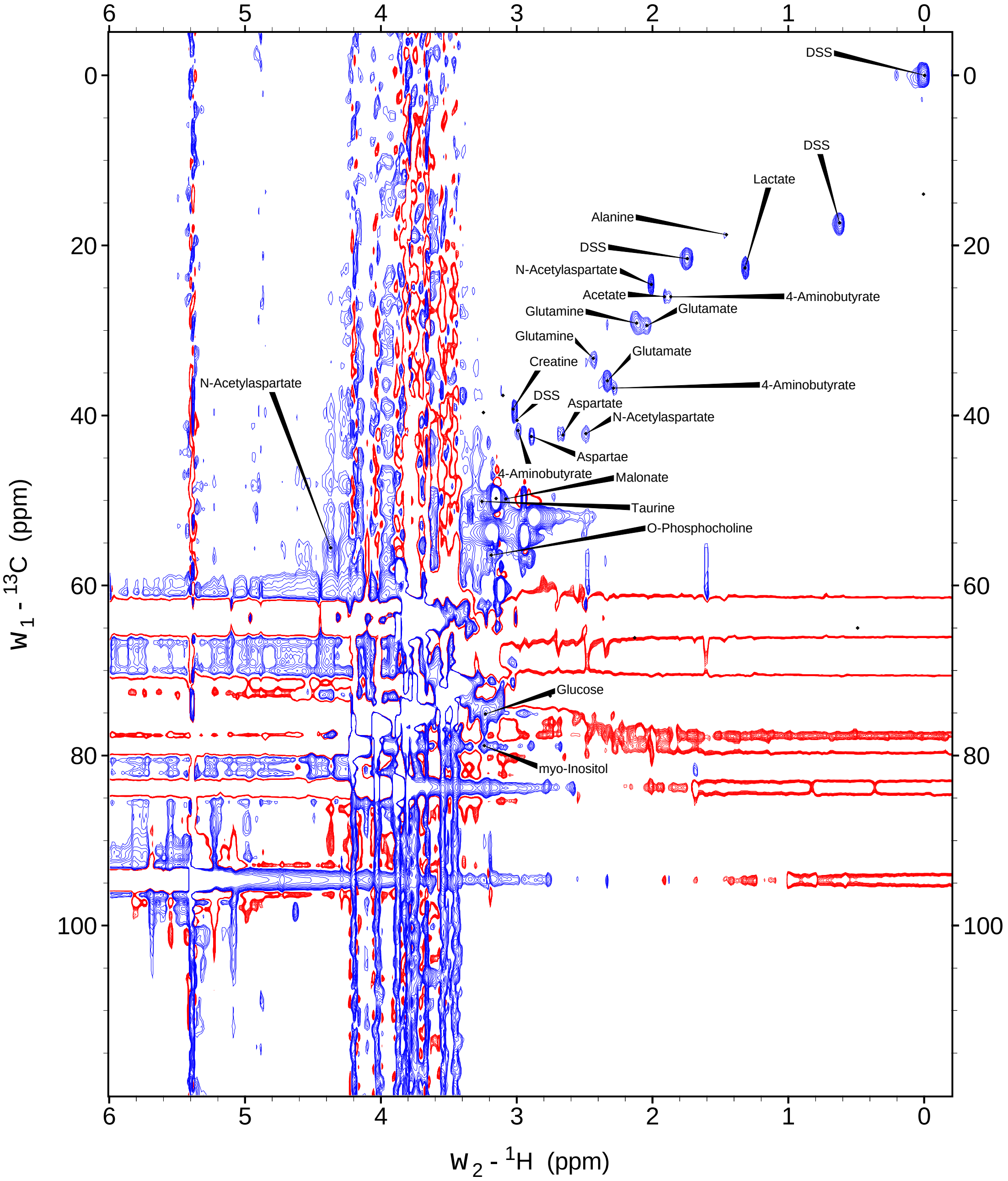

Figure S1. Extended view of Figure 3 showing metabolites identified by the 2D ^{1}H,^{13}C HSQC spectrum of synaptosomes obtained from control animals.

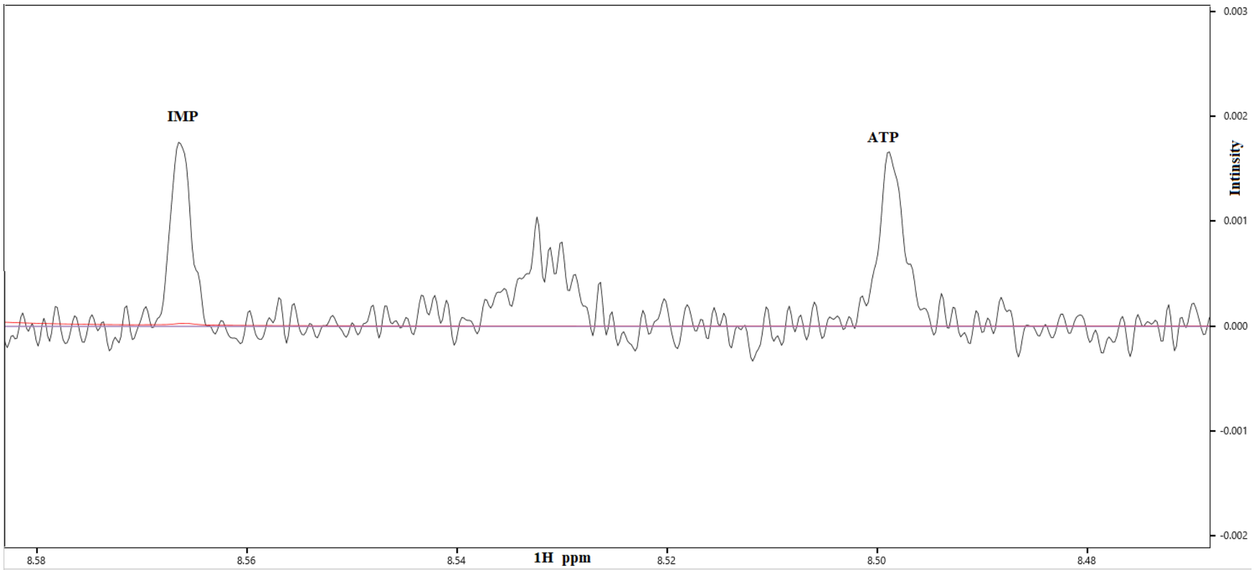

Figure S2. Peaks assigned to IMP and ATP in the 1D spectrum obtained for an animal in the control group can be clearly identified. The peaks for IMP and ATP are the weakest peaks in the spectrum with areas approximately one-half those of the next weakest peaks (succinate and homo-cysteine). The concentrations of ATP and IMP are estimated to be about 5 μM.
